# Application of Red Onion Peel Extract for Green Synthesis of Silver Nanoparticles in Hydrogels Exhibiting Antimicrobial Properties

**DOI:** 10.3390/gels9060498

**Published:** 2023-06-19

**Authors:** Judita Puišo, Algimantas Paškevičius, Jonas Žvirgždas, Todorka L. Dimitrova, Andrejus Litvakas, Diana Adliene

**Affiliations:** 1 Department of Physics, Kaunas University of Technology, Studentų Str. 50, LT-51368 Kaunas, Lithuania; andrejus.litvakas@ktu.edu; 2 Laboratory of Biodeterioration Research, Institute of Botany, Nature Research Centre, Akademijos Str. 2, LT-08412 Vilnius, Lithuania; algimantas.paskevicius@gamtc.lt (A.P.); jonas.zvirgzdas@gamtc.lt (J.Ž.); 3 Faculty of Physics and Technology, University of Plovdiv “Paisii Hilendarski”, Tzar Assen Str. 24, 4000 Plovdiv, Bulgaria; doradimitrova@uni-plovdiv.bg

**Keywords:** red onion peel, gels, green synthesis, Ag NPs, UV-Vis spectrophotometry, antimicrobial activity

## Abstract

UV-initiated green synthesis of metal nanoparticles by using plant extracts as photoreducing agents is of particular interest since it is an environmentally friendly, easy-to-maintain, and cost-effective method. Plant molecules that act as reducing agents are assembled in a highly controlled way which makes them suitable for metal nanoparticle synthesis. Depending on the plant species, their application for green synthesis of metal nanoparticles for diverse applications may contribute to the mediation/reduction in organic waste amounts, thus enabling the implementation of the circular economy concept. In this work, UV-initiated green synthesis of Ag nanoparticles in hydrogels and hydrogel’s thin films containing gelatin (matrix), red onion peel extract of different concentrations, water, and a small amount of 1 M AgNO_3_ have been investigated and characterized using UV-Vis spectroscopy, SEM and EDS analysis, XRD technique, performing swelling experiments and antimicrobial tests using bacteria (*Staphylococcus aureus*, *Acinetobacter baumannii*, *Pseudomonas aeruginosa*), yeasts (*Candida parapsilosis*, *Candida albicans*) and microscopic fungi (*Aspergillus flavus*, *Aspergillus fumigatus*). It was found that the antimicrobial effectiveness of the silver-enriched red onion peel extract–gelatin films was higher at lower AgNO_3_ concentrations as compared to those usually used in the commercially available antimicrobial products. The enhancement of the antimicrobial effectiveness was analyzed and discussed, assuming the synergy between photoreducing agent (red onion peel extract) and silver nitrate (AgNO_3_) in the initial gel solutions leading to the intensification of Ag nanoparticles production.

## 1. Introduction

Onion (*Allium cepa* L.) is an important foodstuff consisting of phytomolecules such as phenolic acid, flavonoids, copaenes, thiosulfinate, organosulfur compounds, and anthocyanin [1,2,3,4,5,6,7], which initiate protective effects against different degenerative pathologies such as cardiovascular and neurological diseases, cancer and other dysfunctions based on oxidative stress [2]. 

Flavonoids are the largest group of phenolic compounds that account for most of the antioxidant activity in onions. At least 25 different flavonols have been determined in onion, but quercetin derivatives are the most important ones in all onion cultivars (accounting for about 80–95% of total flavonols) [8]. The quantitative content of anthocyanins in some red onion cultivars may reach approximately 10% of the total flavonoid content [8]. It was also shown [6,7,8,9,10,11,12,13,14] that the peel of onion bulbs is richer in flavonols and anthocyanins, e.g., the dry skin of red onion contains ~63% of the total amount of anthocyanins that are present in bulbs. 

It could be mentioned that stress conditions such as UV light, wounding, or infection induce the biosynthesis of phenolic compounds [9]. Deriving the extract from the non-edible outside layers (skin, peel) of red onion using eco-friendly solvents allows to produce auto adhesive, biocompatible, and pain-free hydrogel films for dermal application [15] and food preservation [16]. Additionally, it should be noted that onion peels were found to be excellent reductants for ZnO [17,18], Fe_2_O_3_ [19], Au [20,21,22], Ag [20,23,24], and Cu [25] nanoparticles synthesis.

Hydrogels are three-dimensional cross-linked networks of hydrophilic polymer chains that have the ability to absorb and retain large amounts of water or biological fluids. Water serves as the medium for hydrogel swelling and for the diffusion of solutes. The amount of water or solvent absorbed by the hydrogel is an important parameter that influences its properties; however, the main component of hydrogel is polymer, which might be synthetic (polyacrylamide polyvinyl alcohol, polyethylene glycol, etc.) and natural (hyaluronic acid, chitosan, and gelatin, agarose, etc.). The specific composition of a hydrogel can be tailored to achieve the desired properties and functionality for a particular application. The choice of polymers, cross-linkers (used to secure gelation and stability of hydrogels), additives (bioactive agents and/or nanoparticles), and other components. Depends on factors such as biocompatibility, mechanical strength, biodegradability, drug release kinetics, and the targeted environment or tissue. 

The use of biocompatible hydrogels ensures that the material is non-toxic and compatible with living tissues. This property is crucial for medical applications, as it minimizes adverse reactions and promotes tissue integration.

When incorporating plant extracts and silver nanoparticles into biocompatible hydrogels, several potential benefits can be achieved: antimicrobial properties, which can help prevent the growth of bacteria and other microorganisms and reduce the risk of infection; and wound healing properties exhibited by certain plants, which can promote tissue regeneration and accelerate the healing process of wounds. However, it’s important to note that the development and utilization of such hydrogels require extensive research and testing to ensure their safety and efficacy. The choice of plant extracts, silver nanoparticle concentration, and formulation parameters must be carefully optimized to achieve the desired properties and therapeutic effects.

The benefits of plant extract-gelatin-metal NPs composites have been approved for different plants by many authors. It is also known [26] that the effective synthesis of silver (Ag) nanoparticles can be achieved through photochemical reduction processes in hydrogels exposed to UV light. Based on this, it is unclear how it is possible to achieve effective reduction (creation of a significant amount) of metal NPs from salts just using gelatin and plant extracts as reductants without additional application of UV exposure or other methods contributing to NPs reduction [27].

The aim of this work was to produce and characterize Ag NPs enriched antimicrobial and recyclable gel films containing red onion peel extract for possible medical and food packaging applications, implementing in parallel the principles of green policy in the circular economy.

## 2. Results and Discussion

### 2.1. Red Onion Peel Extract–Gelatin Gels with Silver Particles

**Figure 1:**
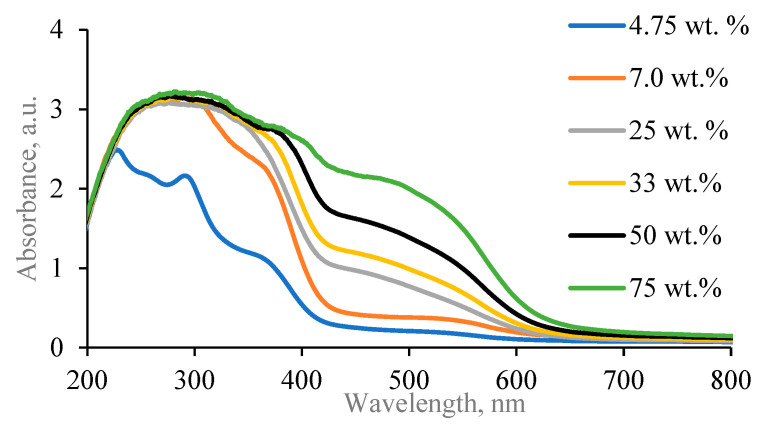
UV-Vis absorbance spectra of diluted red onion peel extract solutions.

It is known [28,29,30,31] that onion peel extract absorbance peak and silver nanoparticles plasmon resonance peak positions are in the same wavelength interval and may overlap. To avoid that and to reduce the impact of onion peel extract on the identification of surface plasmon resonance position of synthesized Ag NPs in experimental hydrogels, dilution of onion peel extract in distilled water was undertaken, creating different concentrations of the extract water solutions and the UV-Vis spectra of the diluted onion peel extract solutions were investigated (Figure 1).

It was found that the red onion extract dilution with distilled water caused a blueshift of the absorbance band. The obtained UV-Vis spectra showed a broad peak with shoulder, indicating the presence of anthocyanins in red onion extract, which were responsible for a high absorbance at 240–280 nm and 465–560 nm [32,33,34] and also flavanols with absorbance peaks at 240–280 nm and 300–380 nm [32]. The pH of red onion peel extract increased from 3.85 (just prepared) to 4.05 (after dilution to 75 wt.%). 

Analysis of UV-Vis spectra and pH led to the suggestion that the most suitable concentrations of red onion peel extract might be between 2.4 wt.% and 7.00 wt.%. 

**Figure 2:**
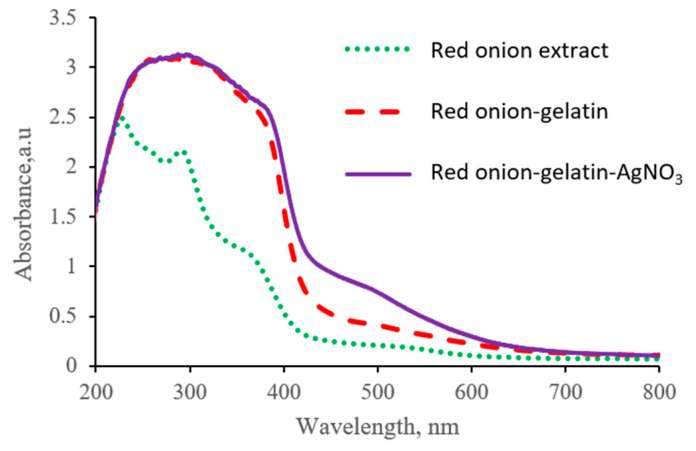
UV-Vis spectra of red onion extract-gel-silver nitrate hydrogels containing 25 wt.% of onion peel extract and its substitutes.

In order to explore this hypothesis, the contribution of each hydrogel’s component to UV-Vis absorbance spectra was examined by separately analyzing the UV-Vis absorbance spectra of the diluted red onion peel extract (4.75 wt.%), red onion extract-gelatin composition, and red onion extract-gelatin-silver nitrate hydrogel (Figure 2). It should be noted that the usage of 9.5 wt.% of gelatin was based on our previous experience [31].

It was possible to distinguish the absorbance peak at 488 nm, which corresponds to the position of the LSPR peak of Ag particles, thus indicating possible biosynthesis of Ag nano seeds in the initial gel-red onion peel extract-AgNO_3_ mixture also without application UV exposure. Synthesis of Ag NPs propagates via cationic silver (Ag^+^) interaction with flavonoids (at least quercetin) and polyphenols that are present in the red onion peel, as it was indicated in [35]. However, it should be noted that the amount of the reduced metallic silver was rather low and not sufficient for medical applications. 

Based on the performed examination, the following composition of hydrogel was considered for further investigation: red onion extract (4.75 wt.%), gelatin (9.50 wt.%), silver nitrate (0.039 wt.%), and water.

It is known [31,36] that the prolonged UV exposure of water-based solutions is related to the production of a large number of hydrated electrons. It is also known [31] that UV exposure usually induces the defragmentation of biological compounds, such as red onion peel extract, to free radicals. Both hydrated electrons and free radicals may reduce silver cations of AgNO_3_ into silver nanoparticles. Therefore, the selection of the appropriate concentrations of red onion peel extract (unused bio-waste) and silver nitrate in the hydrogel’s composition combined with the UV exposure allows for controlling the size and quantity of the produced Ag-NPs. 

**Figure 3:**
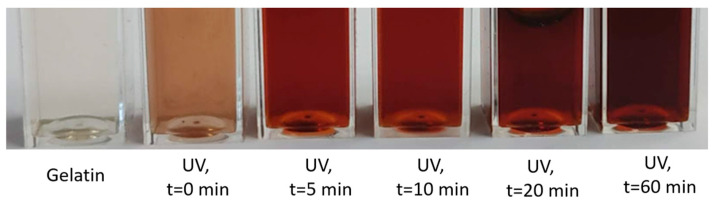
Photographs of differently exposed cuvettes filled with onion peel extract-gelatin-silver nitrate hydrogels and gelatin.

In order to produce the maximum amount of silver nanoparticles in red onion peel-extract-gelatin-silver nitrate hydrogels, experimental samples were additionally exposed to UV (36W), varying exposure time from 0 to 60 min. Significant color changes were observed in the exposed gel samples. These color changes were dependent on red onion peel extract concentration, the concentration of added silver nitrate (source of the produced nanoparticles), and UV exposure time (Figure 3).

**Figure 4:**
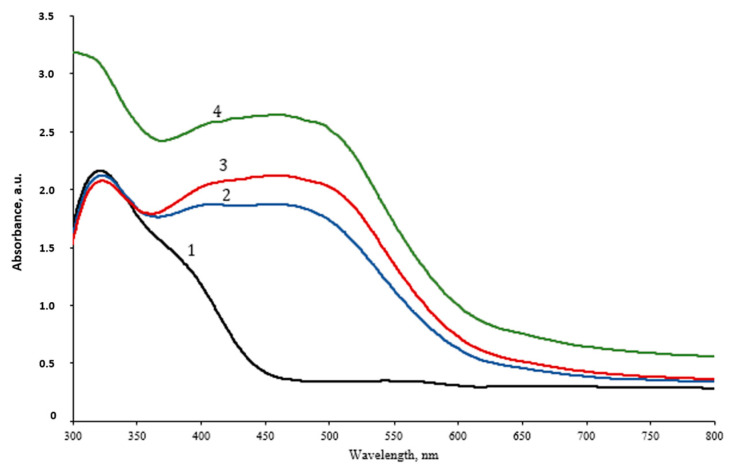
UV-Vis spectra of the UV-exposed red onion peel-gelatin-silver nitrate hydrogels indicating the creation of Ag NPs: 1—not exposed gel; 2—5 min exposed gel; 3—10 min exposed gel; 4—20 min exposed gel.

The UV-Vis spectra of the UV-exposed hydrogel samples (Figure 4) indicated two absorption peaks: a peak at 335 nm which was visible due to possible aggregation of initially reduced silver seeds, and a broad absorption band between wavelengths of 367 nm and 539 nm, with the average maximum peak value at 459 nm which was integral for both: Ag NPs reduced by red onion peel extract and Ag NPs formed in experimental hydrogels due to UV exposure. The intensity of the peak was slightly higher for longer exposures, thus confirming the increasing amount of photosynthesized silver nanoparticles of various sizes remained broad. It should be noted that some changes in hydrogel’s structure upon UV exposure might also be present due to the fact [37] that even physically stable composite hydrogel contains a certain portion of sol (~40% usually). The slight decrease in the gel fraction might be observed due to the reduced cross-linking in hydrogel via competing interactions of NPs with pedant groups in gelatin. In the case of NPs agglomeration, which limits the number of particles in the gel-particle interface, a small increase in gel fraction is possible. Since the amount of AgNO_3_ was very small (0.039 wt.%) in the hydrogel, it was suggested that some growth of NPs was present in experimental samples. This suggestion was experimentally approved when analyzing EDS results obtained for the hydrogel films.

### 2.2. Red Onion Peel–Gelatin Films Containing Silver Nanoparticles

**Figure 5:**
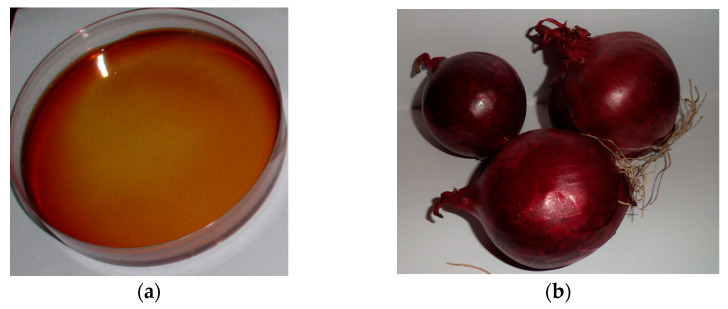
A photograph of red onion peel extract–gelatin-silver nitrate gel films in a Petri dish (**a**) and read onion bulbs (**b**).

An example of experimentally prepared and 20-min-exposed red onion–gelatin film containing silver nanoparticles is presented in Figure 5.

#### 2.2.1. UV-Vis Analysis 

**Figure 6:**
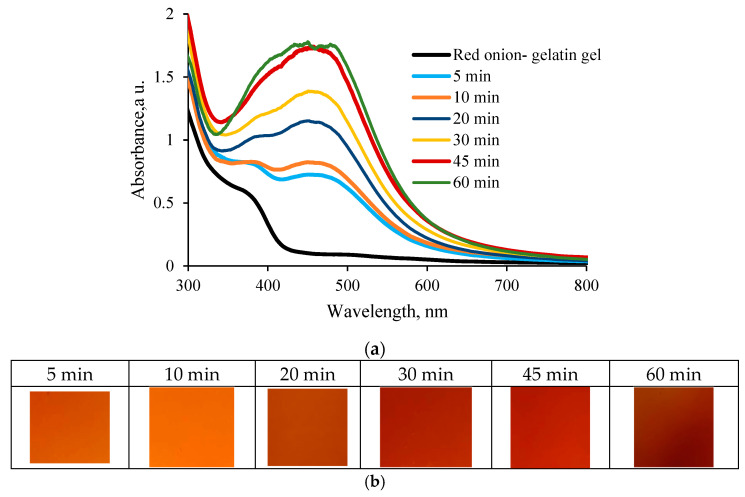
UV-Vis spectra of the exposed red onion peel–gelatin-AgNO_3_ films indicating formation of Ag (**a**) and color changes of hydrogel films after UV exposure (**b**). UV-Vis Spectrum of red onion-gelatin *gel* is provided for comparison.

Performed investigation of the UV-exposed hydrogel films revealed that the corresponding absorbance peaks, which might indicate the presence of synthesized AgNPs (LSPR peaks of AgNPs), were clearly seen in the measured UV-Vis spectra at the wavelength of 457.6 nm, and the color of films was gradually changing with the increased exposure duration (Figure 6).

**Figure 7:**
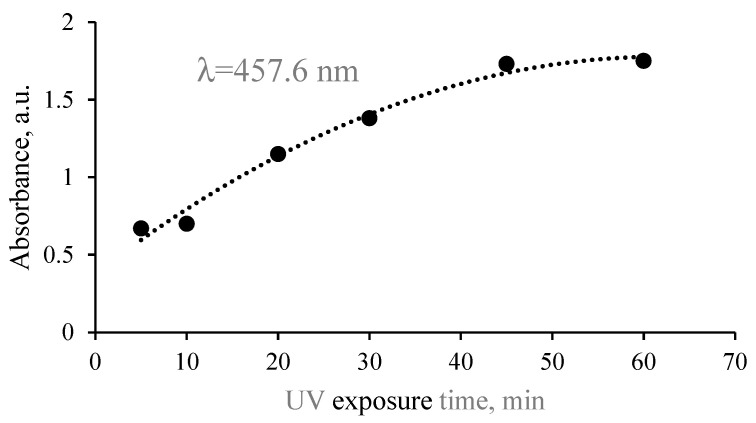
Exposure-duration-related intensity variations of the LSPR absorbance in red-onion-peel–gelatin films continuing photosynthesized Ag nanoparticles.

As in the previous case with hydrogel solution, prolonged exposure of the hydrogel films was responsible for the absorbance band broadening; however, the absorbance bands of hydrogel films were narrower, and the LSPR peak of created Ag NPs was better expressed as compared to those measured for the hydrogel solutions. This was explained by the fact that prolonged exposure of hydrogel films having reduced amounts of water after drying may create a number of differently sized particles, but their mobility is restricted by the gelatin matrix. Stabilization of silver nanoparticles is realized by the amine pendant groups of the gelatin backbone. Long-term UV exposure of gels may also induce structural changes in the gelatin, which reduces the gelatin’s contribution to the absorbance spectrum of the exposed media [38,39] and, in our case, allows for more clear indication of the LSPR peak intensity (Figure 7).

Since the shape of UV-Vis spectra accounts for the presence of all hydrogel components, the formation and behavior of Ag NPs in the experimental films have been additionally approved by analyzing the results of XRD measurements and EDX measurements, and SEM images of the experimental samples. Supportive information could be obtained from FTIR measurements. 

#### 2.2.2. XRD Measurements

**Figure 8:**
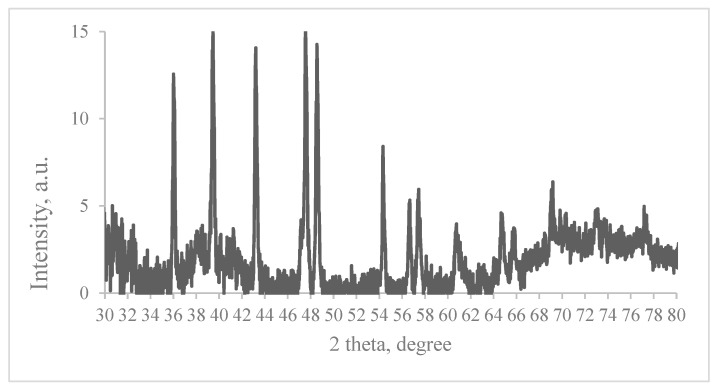
XRD pattern of red-onion-extract-gelatin-AgNO_3_ film after 20 min UV exposure.

An example of the XRD pattern of the red onion peel extract–gelatin–AgNO_3_ film after 20 min UV exposure is provided in Figure 8. The pattern has been compared with the standard powder diffraction card of JCPDS, silver file No. 04-0783. Four peaks at 2θ values of 39.458, 43.208, 64.746, and 77.165 degrees in the experimental diffractogram have been identified as being attributable to the typical (111), (200), (220), and (311) planes of the metallic silver nanoparticles having a face-centered cubic crystalline structure. Other peaks in the diffractogram at 36.008, 47.557, 48.547, 54.337 and 57.647, and 57.426 degrees were related to AgNO_3_, indicating that some amount of this material might not have been reduced and hence remained in the sample. XRD pattern of gelatin is usually seen in the range up to 30 degrees (2θ range) and does not overlap with metallic silver peaks; thus, this part of XRD spectra was omitted in this paper. It should be noted that the experimental XRD results were close to those obtained by other authors [39,40]. 

#### 2.2.3. SEM and EDX Measurements

**Figure 9:**
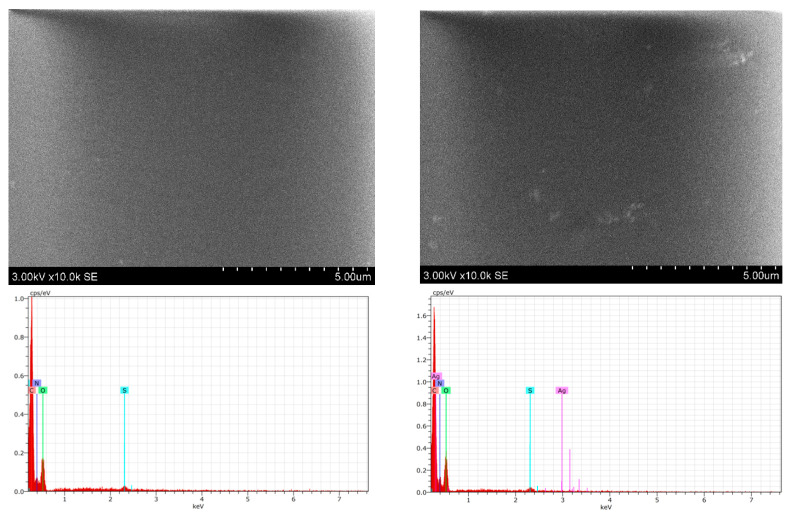
SEM images and sample composition (EDX measurement): not irradiated hydrogel films (R); UV-exposed (20 min) hydrogel films (L).

It is known [37] that 3D network structures with smooth surface morphology are usually formed in hydrogels, as could be seen in the provided SEM images of the experimental samples (Figure 9). This relies on the fact that the UV exposure initiates some restructuring of gelatin which allows for attaching gelatin to the NPS surface via hydrogen bonds and thus reduces the possibility of Ag NPS appearing on the film’s surface. This also fits well with the requirement that the biocompatible films applied for medical purposes should be of reduced toxicity. It should be pointed out that the initial amount of AgNO_3_ in the experimental hydrogel films was very small (0.039 wt.%). 

It was not possible to draw conclusions regarding the amounts of silver nanoparticles in the hydrogel films from the obtained SEM images. A more detailed investigation of the elemental composition of UV-exposed samples performed using the EDX technique (Table 1) revealed that the initial amount of Ag NPs in non-irradiated hydrogel films containing red onion peel extract were below the detection limit. However, it was clearly seen that the amount of metallic Ag was slightly increasing with the prolonged exposure.

#### 2.2.4. Swelling Behavior

**Figure 10:**
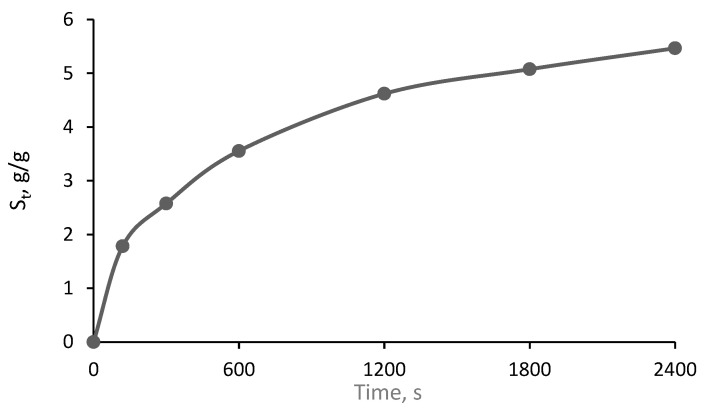
Swelling capacity of red onion-gelatin solution with silver nanoparticles (t = 20 °C).

The swelling ability of the films that are thought to the application as an antimicrobial wound dressing is of crucial importance. An ideal wound dressing should be able to absorb wound exudate and provide a wet environment for the wound. It should be noted [41] that the electrostatic repulsion of the ionic charges of hydrogel’s network is responsible for the gel’s swelling. This process leads to the blockage of the polymer chains, reduction in cross-linking density, and expansion of hydrogels. Since differently sized Ag NPs were formed in the experimental hydrogel films, it was expected [42,43] that the swelling ability of experimental gel films would not have a linear increasing tendency with time. Performed evaluation has shown no linear, time-dependent swelling behavior in the red-onion-gelelatin-film containing silver nanoparticles, indicating a relative high swelling rate (Figure 10).

It was found that the swelling ratio was higher than 60, which is sufficient for the application as hydrogel wound dressing was achieved 30 min after immersion of the hydrogel film in the distilled water. 

### 2.3. Antimicrobial Activity of Red Onion Skin Extract Gels and Films with Silver Nanoparticles

Performed antimicrobial activity assessment in experimental hydrogels has shown (Table 2) that red onion peel-extract-gelatin hydrogels containing Ag NPS were the most effective against *Staphylococcus aureus*, *Acinetobacter baumannii*, *Pseudomonas aeruginosa*, and *Pseudomonas aeruginosa* bacteria. Significantly lower and very similar antimicrobial effect of experimental hydrogels was observed on *Candida parapsilosis* and *Candida albicans* yeasts, but there was no effect on *Candida albicans* observed in the case when hydrogel film containing Ag NPs was applied. The microscopic fungus, *Aspergillus flavus*, was completely unaffected by experimental hydrogels.

**Figure 11:**
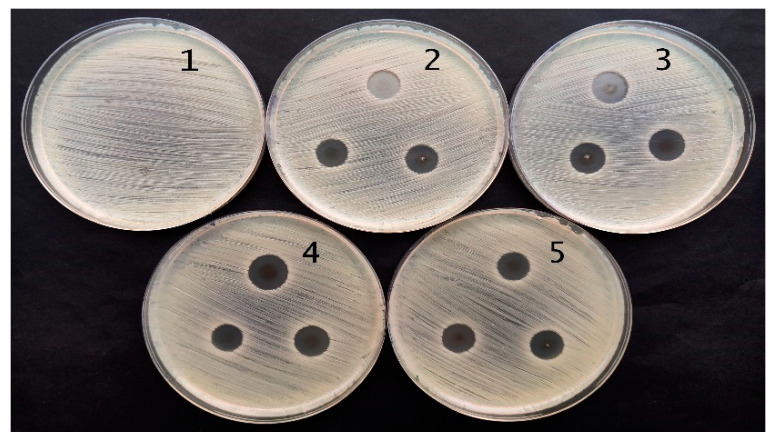
Antimicrobial activity of red onion peel extract hydrogels containing silver nanoparticles against *Pseudomonas aeruginosa bacteria ATCC 15442*: (1) control; (2) 5 min exposed gel; (3) 10 min exposed gel; (4) 20 min exposed gel; (5) 60 min exposed gel.

**Figure 12:**
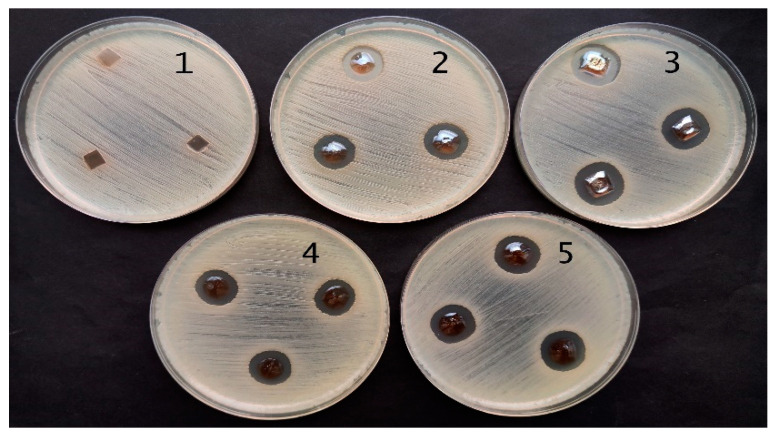
Antimicrobial activity of red onions peel extract hydrogel films containing silver nanoparticles against *Pseudomonas aeruginosa bacteria ATCC 15442*: (1) control; (2) 5 min exposed film; (3) 10 min exposed film; (4) 20 min exposed film; (5) 60 min exposed film.

The antimicrobial effect was slightly higher for hydrogel films, as could be seen from the results obtained for *Pseudomonas aeruginosa bacteria ATCC 15442* which are provided in Figure 11 and Figure 12. The effect was dependent on the amount of Ag NPs in hydrogel samples which correlated with the duration of hydrogel’s UV exposure. 

It was found that dried hydrogel films containing red onion peel extract, gelatin, and Ag NPs obtained via green photosynthesis were indicating higher [32,44,45,46] antimicrobial activity as compared to hydrogels containing different plant extracts.

## 3. Conclusions

Nanosilver-gelatin-red-onion extract composite hydrogels were produced applying UV exposure supported green synthesis method and characterized using common analytical methods. The synthesized nanoparticles were differently sized and exhibited face-centered cubic crystalline structure, fixed inside of a gelatin matrix. 

It was found that hydrogels containing 4.75 wt.% of red onion peel extract, 9.5 wt.%, and 0.039 wt.% of 1 M AgNO_3_ water solution were suitable for the formation of free-standing gel films that may produce a sufficient amount of silver nanoparticles upon UV exposure which is needed to secure antimicrobial efficiency of films. 

Experimental hydrogel films indicated relatively high swelling behavior achieving a swelling ratio > 60, which is sufficient for wound dressing 30 min after their immersion in distilled water.

Both hydrogels and free-standing gel films were most effective against *Staphylococcus aureus*, *Acinetobacter baumannii*, and *Pseudomonas aeruginosa* bacteria; however, the antibacterial efficiency of red onion hydrogel films enriched with Ag NPs was higher, as compared to hydrogel solution of the same composition due lower mobility of silver ions in the media with reduced amount of water and fixation of synthesized Ag NPS in gelatin matrix.

## 4. Materials and Methods

### 4.1. Preparation of Silver Nitrate-Red Onion Extract-Gelatin Hydrogels 

The peels of fresh red onion (*Allium cape var. cepa ‘Red Karmen’*) were removed from the bulbs and dried. A total of 2.65 g of dried red onion peel was immersed in 53 g of distilled water and heated for 20 min at 62.5 °C. The prepared extract was filtered twice using a white-band filter (Filtrak, Germany, size of pores 8–12 µm) and was ready to use. For the hydrogel preparation, a certain amount of 1M AgNO_3_ water solution was dropped into red onion extract and then admixed to the dissolved gelatin under continuous stirring. Silver nitrate powder (AgNO_3_, CAS No 7761-88-8, purity ≥ 99.9%, Sigma–Aldrich, Poznan, Poland) and gelatin from porcine skin (250 Bloom, CAS 9000-70-8, Sigma–Aldrich) were used for the formation of hydrogels. In order to decide the optimal concentration of the red onion peel extract in silver nitrate–red onion-gelatin hydrogel, different read onion extract concentrations (4.75 wt.%, 7 wt.%, 25 wt.%, 33 wt.%, 50 wt.%, and 75 wt.%), which were obtained diluting onion extract in the corresponding amount of water, were used for gel production. This was absolutely necessary, taking into account that the absorbance peak of the red onion peel extract appears in the wavelength region [36] where the plasmon resonance peak of synthesized silver nanoparticles may be observed. In order to avoid agglomeration of the photosynthesized silver nanoparticles, a silver nitrate concentration of 0.039 wt.% in the gel was selected according to our previous experience [37,38,39]. Several batches of gel samples with various concentrations of onion peel extract have been prepared. Prepared gels were poured into semicro (1.5–2.5 mL) PMMA cuvettes (METRIA) and stored in darkness for 24 h to settle.

### 4.2. Formation of Silver Nitrate Red Onion Extract-Gelatin Films

5 g of the red onion peel extract-gelatin-silver nitrate hydrogels of the experimentally adjusted composition that were cast in plastic Petri dishes (ø = 55 mm) and then dried in the open air in a dark box at room temperature (22 ± 2 °C) for 24 h to form the films. 

It was found that the average mass loss in onion peel extract-gelatin films containing Ag nanoparticles was 8.6–10.13 wt.% of during the drying process. The thickness of the dried films prepared from 5 g of gels was (142.6 ± 11.3) µm. 

### 4.3. UV Exposure of Experimental Samples

Photoreduction of silver ions in hydrogels and in gel films was performed under UV exposure using UV lamps (power-36 W, max UV emission peak at 365 nm, and weak blue light emission peak at 404 nm). The exposure time of samples varied from 0 to 60 min. 

### 4.4. Instruments and Methods for Hydrogel Investigation

Active acidity of primary extracts (control solutions) and extracts with synthesized silver nanoparticles was measured by HI 98103Checker^®^ (pH Tester, pH meter, measurement range 0–14 pH, resolution ±0.1 and accuracy ±0.2 Hanna Instruments, Inc., Woonsocket, RI, USA). Optical properties of experimental samples were evaluated by Jasco V 650 spectrometer (JASCO Corporation, Tokyo, Japan) working in absorbance photometric mode with a measurement range from 200 nm to 800 nm, UV-Vis bandwidth 0.1 nm; and UV-Vis photospectrometer Ocean Optics USB 4000 UV-Vis (Ocean Optics, Inc., Orlando, FL, USA) with a measurement range from 200 nm to 1100 nm, UV-Vis bandwidth 1.5 nm. The thickness of the gel films was measured with a digital micrometer (measurement range 0–25 mm, resolution ± 2 µm, accuracy ± 4 µm). 

The surface morphologies of red-onion-gelatin films without and with silver nanoparticles were investigated by scanning electron microscope (SEM, Hitachi S-3400 N, Tokyo, Japan) using a secondary electron detector. 

Elemental mapping of red onion gelatin films without and with silver nanoparticles was performed using energy-dispersive X-ray spectroscopy (EDS, Bruker Quad 5040, Hamburg, Germany).

The structure of synthesized Ag NPS was investigated using a D8 Discover X-ray diffractometer (Bruker AXS GmbH, Karlsruhe, Germany) operating at 40 kV and 40 mA with Cu Kα source (λ = 1.5418 Å) and parallel beam geometry with 60 mm Göbel mirror. Diffraction patterns were recorded using a fast-counting LynxEye detector with an opening angle of 2.475 deg. Additionally, it had a slit opening of 6 mm [32].

Swelling studies of the dried films were carried out in distilled water. The weighed amount of the dried film was immersed in 10 mL of the swelling solution at room (20 °C) temperature. At predefined time intervals, the film was separated from the swelling solution, gently wiped with filter paper, and weighed. The swelling capacity (M) of the films was calculated according to Equation (1) [47,48]:

(1) $$S_{t}=\frac{w_{t}-w_{0}}{w_{0}}$$

where $w_{t}$ was the mass of the film in the swollen state and $w_{0}$ was the initial mass of the dry film. 

### 4.5. Microorganisms and Inoculum Preparation

Bacteria–Staphylococcus aureus ATCC 29213, Acinetobacter baumannii BAA 747, Pseudomonas aeruginosa ATCC 15442, yeasts–Candida parapsilosis CBS 8836, Candida albicans ATCC 90028 and microscopic fungi–Aspergillus flavus BTL G-33, Aspergillus fumigatus BTL G-38 were used in assays. Microorganisms were stored in a −70 °C freezer at the Laboratory of Biodeterioration Research of the Nature Research Centre (Vilnius, Lithuania). Bacteria for antimicrobial tests were grown on nutrient agar (Oxoid, Basingstoke, UK), and yeasts and fungi were grown on Sabouraud dextrose agar (Oxoid, Basingstoke, UK). Inoculums were obtained from overnight bacterial cultures grown on nutrient agar at 37 °C. Yeasts were cultured for 3 days, and fungi were cultured for 7 days at 28 °C. The optical density of the microorganism cell suspensions was measured with a spectrophotometer (Thermo Scientific, Waltham, MA, USA) at a wavelength of 610 nm. The resulting microorganism suspensions were vortexed for 15 s.

### 4.6. Evaluation of the Antimicrobial Activity of the Red Onions Extracts Gels and Films Containing Silver Nanoparticles

The antimicrobial activity of red onion skin extract gels and films containing silver nanoparticles was tested using the agar diffusion method. For the agar diffusion assay, 100 µL of each microorganism suspension was uniformly spread on Mueller–Hinton agar (bacteria) or on Sabouraud dextrose agar (yeasts and microscopic fungi) in 90 mm Petri dishes. The red onion gels (10 µL) and films (5 × 5 mm) containing silver nanoparticles were placed on the surface of each media as the microbial suspension absorbed into it. The plates were incubated at 28 ± 1 °C for 3 (yeasts) or 5 days (microscopic fungi), while the plates with bacteria were incubated at 37 ± 1 °C for 2 days. All tests were triplicated for all strains. The red onion skin extract, gel, and film were used as negative controls. The diameters of the inhibition zones were measured in millimeters after the incubation period. Data were collected and processed with the corresponding program package. Mean, standard errors, and confidence intervals were calculated.

## Figures and Tables

**Table 1:** The elemental concentration of the exposed red-onion-peel–gelatin with Ag nanoparticles.

Symbol	Series	UV = 0 min		UV = 5 min		UV = 10 min		UV = 20 min		UV = 60 min	
		Atom. C [at. %]	Error [%]	Atom. C [at. %]	Error [%]	Atom. C [at. %]	Error [%]	Atom. C [at. %]	Error [%]	Atom. C [at. %]	Error [%]
C	Kα	39.48	10.90	38.81	10.50	38.45	10.20	38.56	10.60	37.30	10.20
O	Kα	33.70	13.40	34.03	12.80	34.29	13.50	34.03	13.30	34.68	13.50
N	Kα	26.31	10.00	26.46	9.30	26.54	10.00	26.63	9.90	27.27	10.00
S	Kα	0.5	0.10	0.45	0.10	0.46	0.10	0.47	0.10	0.40	0.10
Ag	Lα	-	-	0.25	0.10	0.28	0.20	0.31	0.20	0.34	0.20

**Table 2:** Antimicrobial activity of the red-onion-peel-extract hydrogels and films containing silver nanoparticles.

Bacteria	Red Onion Peel-Gelatin	Red Onion Peel Extract	Exposure Duration Related Antimicrobial Activity of Hydrogels				Exposure Duration Related Antimicrobial Activity of Hydrogel Films			
			5 min	10 min	20 min	60 min	5 min	10 min	20 min	60 min
	Size of the Inhibition Zone, mm									
*Staphylococcus aureus* ATCC 29213	8.0 ± 0.0	0	11.3 ± 0.6	12.7 ± 0.6	11.0 ± 0.0	10.7 ± 0.6	14.0 ± 0.0	14.0 ± 0.6	15.3 ± 0.6	15.7 ± 0.6
*Acinetobacter baumannii* BAA 747	6.0 ± 0.0	0	10.7 ± 0.6	11.0 ± 0.0	11.0 ± 0.0	11.0 ± 0.0	12.3 ± 0.6	13.0 ± 0.6	13.3 ± 0.6	12.0 ± 0.0
*Pseudomonas aeruginosa* ATCC 15442	0	0	11.7 ± 0.6	13.0 ± 0.0	13.3 ± 0.6	12.3 ± 0.6	15.7 ± 0.6	18.7 ± 0.6	15.7 ± 0.6	17.3 ± 0.6
*Candida parapsilosis* CBS 8836	0	0	6.0 ± 0.0	6.0 ± 0.0	6.0 ± 0.0	6.0 ± 0.0	6.0 ± 0.0	6.0 ± 0.0	6.0 ± 0.0	6.0 ± 0.0
*Candida albicans* ATCC 90028	0	0	6.0 ± 0.0	6.0 ± 0.0	6.0 ± 0.0	6.0 ± 0.0	0	0	0	0
*Aspergillus flavus* BTL G-33	0	0	0	0	0	0	6.0 ± 0.0	6.0 ± 0.0	6.0 ± 0.0	6.0 ± 0.0
*Aspergillus fumigatus* BTL G-38	0	0	6.0 ± 0.0	6.0 ± 0.0	6.0 ± 0.0	6.0 ± 0.0	6.0 ± 0.0	6.0 ± 0.0	6.0 ± 0.0	6.0 ± 0.0

## Data Availability

Data is available upon request by corresponding authors.
